# Supplementary material for: Degrading permafrost puts Arctic infrastructure at risk by mid-century
Source: Nat Commun. 2018 Dec 11;9:5147. doi: 10.1038/s41467-018-07557-4 (PMC6289964; doi:10.1038/s41467-018-07557-4)
Supplement: Supplementary file 1 — Supplementary Information [file 41467_2018_7557_MOESM1_ESM.pdf]

Supplementary Information
Hjort et al.

Degrading permafrost puts Arctic infrastructure at risk by mid-century

Jan Hjort[1], Olli Karjalainen[1], Juha Aalto[2,3], Sebastian Westermann[4], Vladimir E. Romanovsky[5,6], Frederick E. Nelson[7,8], Bernd Etzelmüller[4], Miska Luoto[2]

[1]*Geography Research Unit, University of Oulu, Oulu, Finland.*
[2]*Department of Geosciences and Geography, University of Helsinki, Helsinki, Finland.*
[3]*Finnish Meteorological Institute, Weather and Climate Change Impact Research, Helsinki, Finland.*
[4]*Department of Geosciences, University of Oslo, Oslo, Norway.*
[5]*Geophysical Institute, University of Alaska Fairbanks, Fairbanks, Alaska, USA.*
[6]*Earth Cryosphere Institute, Tyumen Science Centre, Siberian Branch of the Russian Academy of Science, Tyumen, Russia.*
[7]*Department of Geography, Environment, and Spatial Sciences, Michigan State University, East Lansing, Michigan, USA.*
[8]*Department of Earth, Environmental, and Geographical Sciences, Northern Michigan University, Marquette, Michigan, USA.*

Table of Contents **Page**

Supplementary Figures 2

Supplementary Tables 7

Supplementary Figures

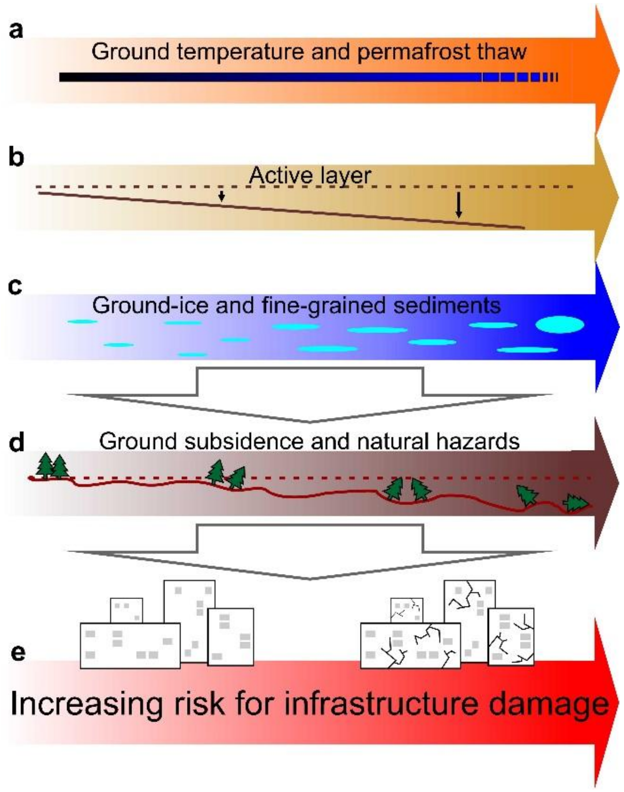

Supplementary Figure 1. Key environmental factors causing ground instability and infrastructure hazards in the permafrost domain[8,9,13,27]. Increasing ground temperature (especially above −3 °C) and thaw of near-surface permafrost (**a**), thickening of active layer (seasonally thawed surface layer atop permafrost) (**b**), or higher ground-ice and fine-grained sediment content (**c**) increase ground instability and natural hazards (e.g., ground subsidence and formation of uneven thermokarst terrain) (**d**), which may lead to loss of structural bearing capacity and damage to human infrastructure (**e**).

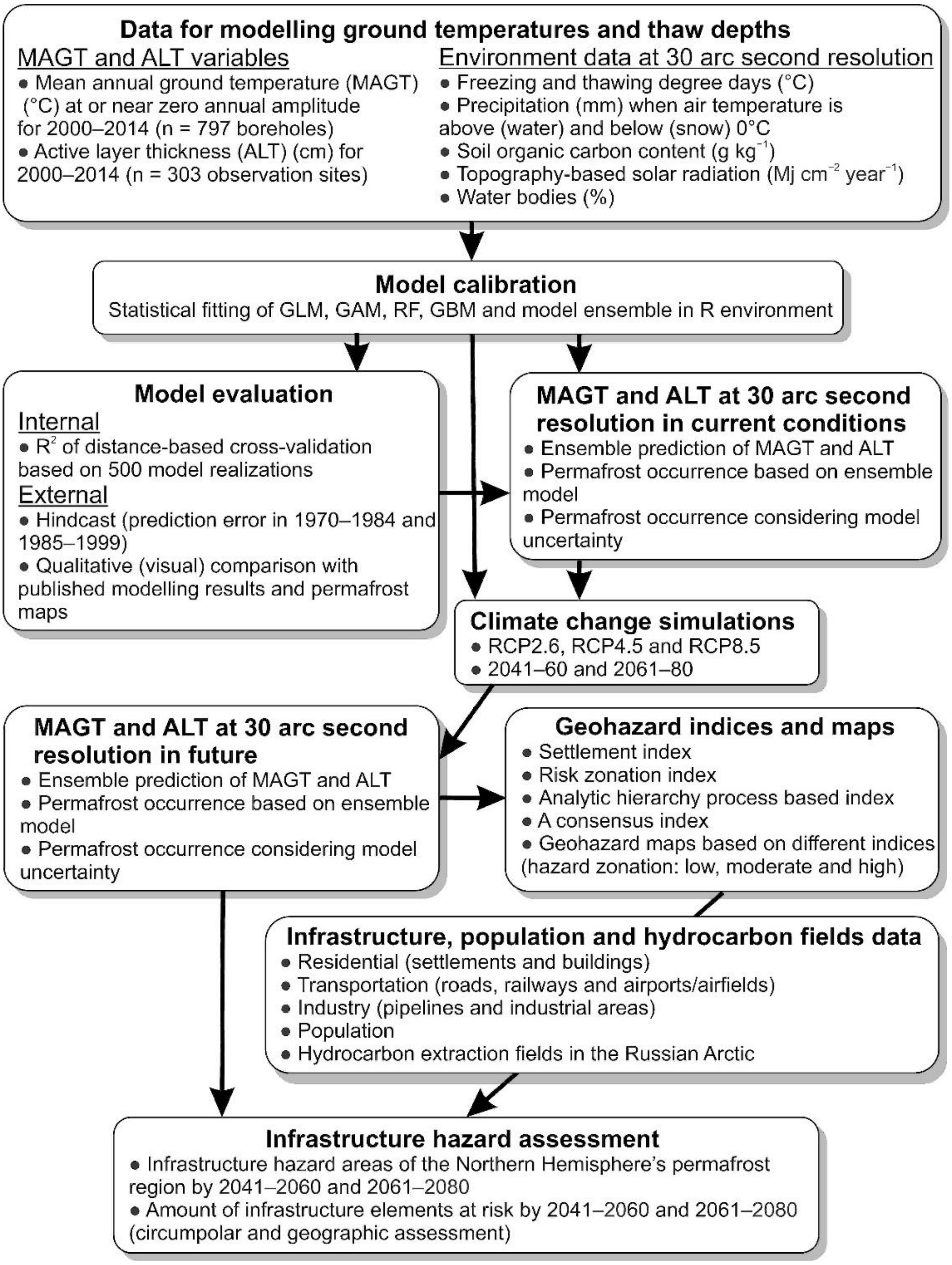

Supplementary Figure 2. Schematic presentation of the study steps. Abbreviations: GLM = generalized linear model, GAM = generalized additive model, RF = random forest, GBM = generalized boosting method, RCP = Representative Concentration Pathways[25].

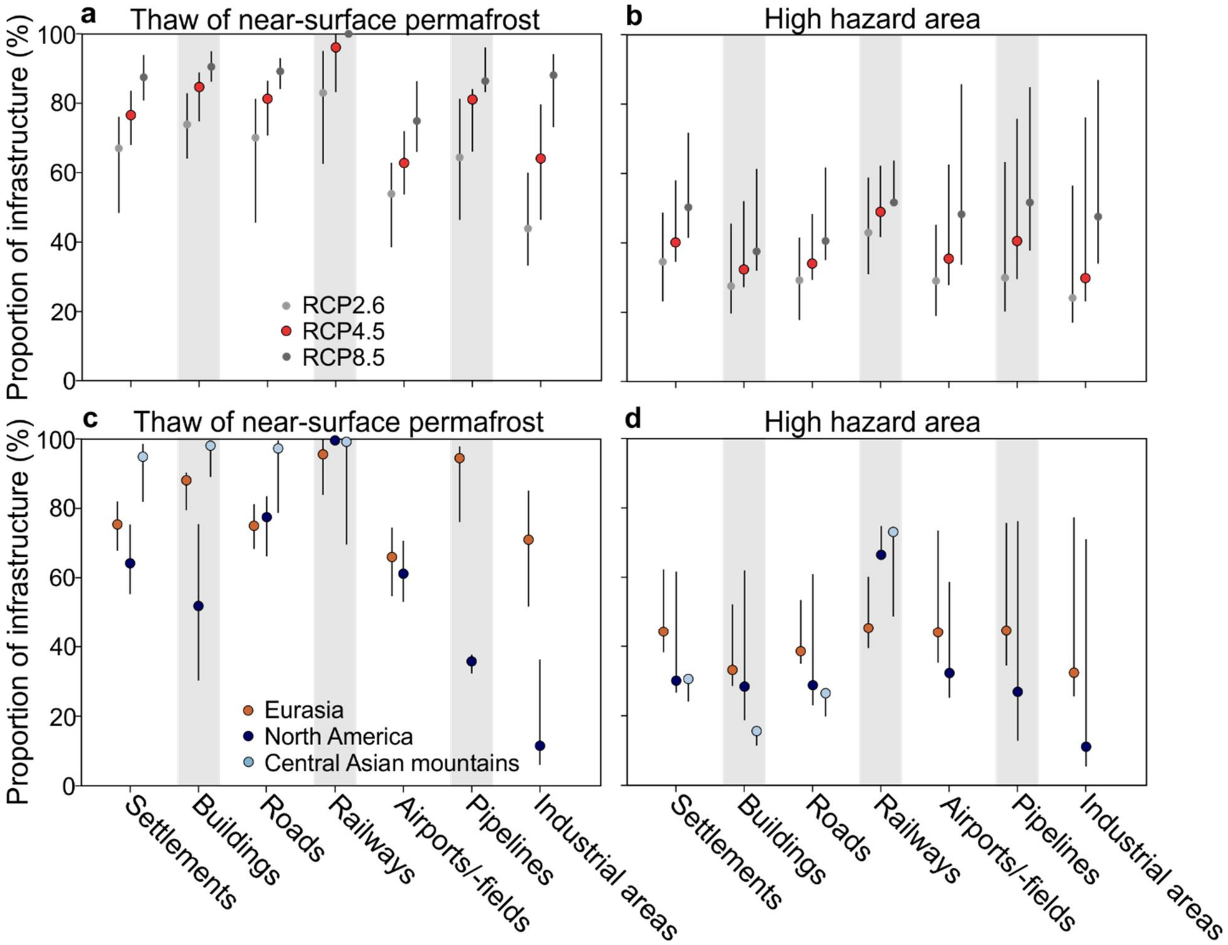

Supplementary Figure 3. Central results of the infrastructure hazard computations by 2070 (2061–2080). Proportion of all residential, transportation, and industrial infrastructure in areas of near-surface permafrost thaw (**a**) and high hazard (**b**) in pan-Arctic permafrost area based on different Representative Concentration Pathway (RCP) scenarios. The comparable results of RCP4.5 for geographical sub-regions (Eurasia, North America and central Asian mountains) are presented in **c** and **d** (also 2061–2080; percentages for airports/-field, pipelines, and industrial areas are not shown for central Asian mountains owing to too few observations). The numerical results are presented in Supplementary Table 3. The uncertainty ranges (bars) are based on the uncertainty in the mean annual ground temperature (**a–d**) and active layer thickness predictions (**b** and **d**).

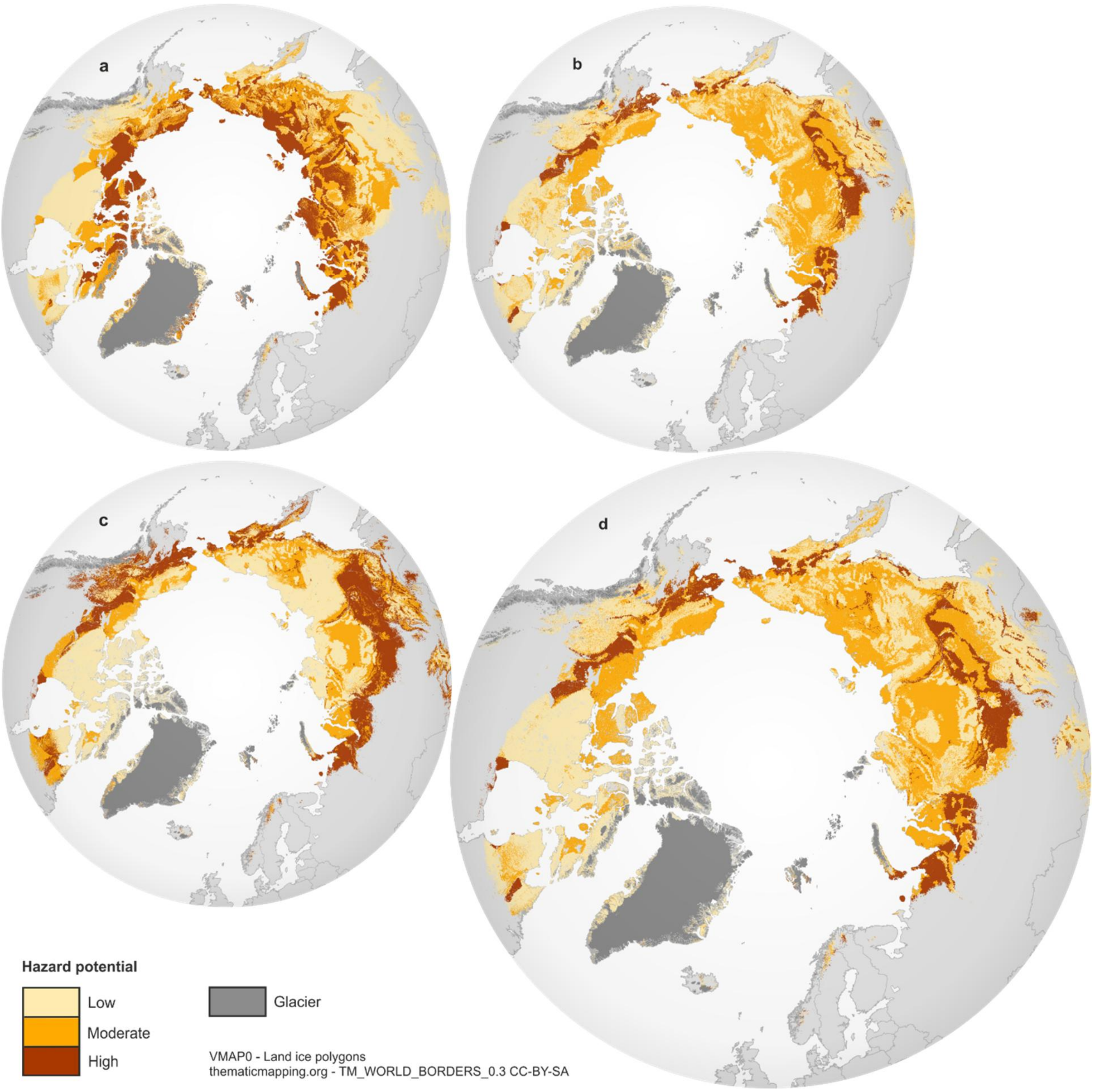

Supplementary Figure 4. Geohazard indices showing hazard potential by risk level for infrastructure damage by the middle of the century (RCP4.5 2041–2060). Settlement index (**a**), risk zonation index (**b**), analytic hierarchy process-based index (**c**), and a consensus of the three indices (**d**) are presented. Note that some of the mid-latitude mountains were excluded to improve the interpretability of the Arctic region. World Borders dataset is from http://thematicmapping.org/downloads/world_borders.php and licensed under CC BY-SA 3.0 (https://creativecommons.org/licenses/by-sa/3.0/).

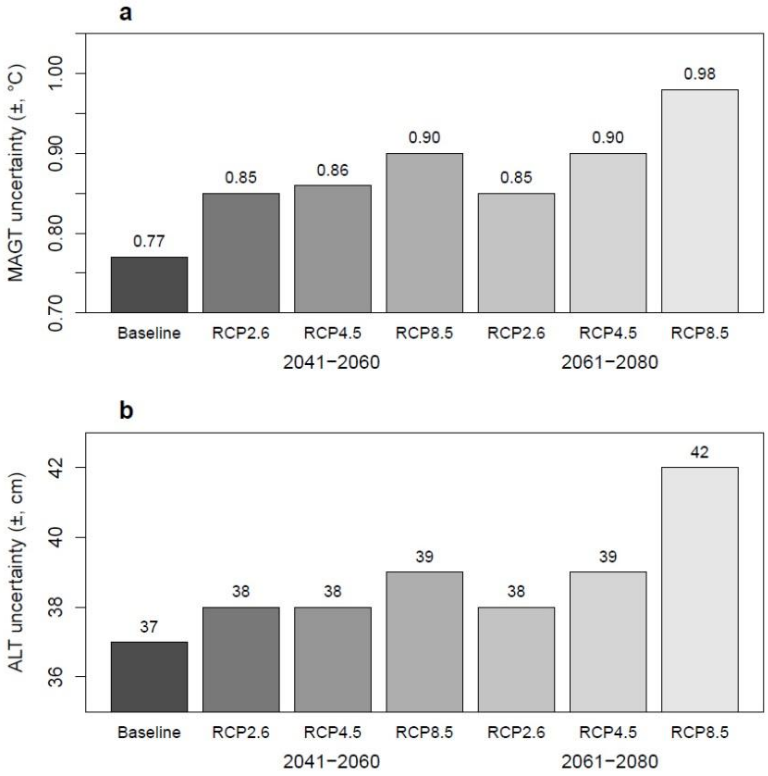

Supplementary Figure 5. Uncertainty of model predictions. Uncertainty ranges for predicted mean annual ground temperature (MAGT) (**a**) and active layer thickness (ALT) (**b**) in baseline (2000–2014) and future climate conditions. Uncertainty was assessed by quantifying the variation of 1,000 ensemble predictions at 100,000 randomly chosen grid cells (glaciers masked out), at each time using 70% random sample of MAGT and ALT observations.

Supplementary Tables

Supplementary Table 1. Numerical results of the specific study targets. The results include data for pan-Arctic population (2015), hydrocarbon extraction fields in the Russian Arctic, major pipelines, and central railways for periods 2041–60 and 2061–80 under three Representative Concentration Pathways (RCPs). Proportion (%) and associated uncertainty range of elements at risk in areas of near-surface permafrost thaw and high hazard (determined by a consensus of geohazard indices, I_c) appear in brackets. The areal coverage of I_c is slightly smaller than that of modelled permafrost owing to the patchiness of sediment property data in certain high-Arctic areas[41].

	Element at risk	On permafrost	In thaw area		In *Ic* area	In high hazard area	
**RCP2.6 2041–2060**	Population	4,906,854	3,093,472	(63.0, 33.3-82.7)	4,567,438	721,308	(15.8, 9.3-30.4)
	Oil/Gas fields in the Russian Arctic	84,170	61,298	(72.8, 51.5-82.5)	83,870	35,143	(41.9, 31.9-71.2)
	TAPS (Prudhoe Bay-Valdez)	914	492	(53.9, 30.5-63.9)	913	291	(31.9, 20.9-57.2)
	Yamal-Nenets gas pipelines	1,341	1,182	(88.2, 54.6-100)	1,341	593	(44.3, 23.5-80.7)
	ESPO (Tayshet-Kozmino)	1,600	1,554	(97.2, 85.6-100)	1,600	733	(45.8, 43.6-46.6)
	Qinghai-Tibet railway (Lhasa-Xining)	510	412	(80.9, 20.6-99.6)	479	379	(79.1, 20.1-99.0)
	Obskaya-Bovanenkovo railway	563	245	(43.4, 8.4-56.8)	563	176	(31.2, 5.8-76.0)
**RCP4.5 2041–2060**	Population	4,906,854	3,612,746	(73.6, 46.3-87.3)	4,567,438	944,995	(20.7, 12.4-33.4)
	Oil/Gas fields in the Russian Arctic	84,170	64,560	(76.7, 61.1-85.4)	83,870	38,004	(45.3, 34.9-77.9)
	TAPS (Prudhoe Bay-Valdez)	914	555	(60.7, 44.8-66.2)	913	293	(32.1, 29.1-66.7)
	Yamal-Nenets gas pipelines	1,341	1,262	(94.1, 69.7-100)	1,341	673	(50.2, 29.5-92.8)
	ESPO (Tayshet-Kozmino)	1,600	1,586	(99.1, 93.8-100)	1,600	733	(45.8, 45.8-46.6)
	Qinghai-Tibet railway (Lhasa-Xining)	510	473	(92.9, 36.6-99.8)	479	438	(91.4, 34.6-99.5)
	Obskaya-Bovanenkovo railway	563	277	(49.2, 18.7-81.8)	563	209	(37.1, 13.5-91.0)
**RCP8.5 2041–2060**	Population	4,906,854	4,120,106	(84.0, 65.5-92.5)	4,567,438	1,061,324	(23.2, 19.7-37.2)
	Oil/Gas fields in the Russian Arctic	84,170	73,070	(86.8, 76.1-94.1)	83,870	48,143	(57.4, 44.4-84.0)
	TAPS (Prudhoe Bay-Valdez)	914	589	(64.5, 56.6-69.7)	913	314	(34.4, 31.7-76.4)
	Yamal-Nenets gas pipelines	1,341	1,341	(100, 90.2-100)	1,341	752	(56.1, 45.7-100)
	ESPO (Tayshet-Kozmino)	1,600	1,600	(100, 98.1-100)	1,600	733	(45.8, 45.8-46.6)
	Qinghai-Tibet railway (Lhasa-Xining)	510	506	(99.3, 66.8-100)	479	470	(98.0, 64.5-99.9)
	Obskaya-Bovanenkovo railway	563	422	(74.9, 45.3-100)	563	406	(72.1, 32.9-100)
**RCP2.6 2061–2080**	Population	4,906,854	3,134,647	(63.9, 34.1-83.3)	4,567,438	765,476	(16.8, 9.9-31.1)
	Oil/Gas fields in the Russian Arctic	84,170	62,281	(74.0, 55.6-83.4)	83,870	35,901	(42.8, 33-76.5)
	TAPS (Prudhoe Bay-Valdez)	914	523	(57.3, 36.5-64.8)	913	292	(31.9, 24.3-62.4)
	Yamal-Nenets gas pipelines	1,341	1,188	(88.6, 59.1-100)	1,341	599	(44.7, 24.4-87.0)
	ESPO (Tayshet-Kozmino)	1,600	1,559	(97.5, 88.1-100)	1,600	733	(45.8, 44.8-46.6)
	Qinghai-Tibet railway (Lhasa-Xining)	510	411	(80.8, 19.4-99.6)	479	378	(79.0, 19.1-99.0)
	Obskaya-Bovanenkovo railway	563	251	(44.5, 9.7-58.5)	563	183	(32.4, 6.7-77.4)
**RCP4.5 2061–2080**	Population	4,906,854	4,108,172	(83.7, 64.8-92.1)	4,567,438	1,052,540	(23.0, 19.4-36.9)
	Oil/Gas fields in the Russian Arctic	84,170	72,037	(85.6, 74.0-92.6)	83,870	47,162	(56.2, 42.3-83.0)
	TAPS (Prudhoe Bay-Valdez)	914	589	(64.5, 56.3-68.5)	913	318	(34.8, 31.7-74.7)
	Yamal-Nenets gas pipelines	1,341	1,341	(100, 86.5-100)	1,341	752	(56.1, 42.1-100)
	ESPO (Tayshet-Kozmino)	1,600	1,600	(100, 98.0-100)	1,600	733	(45.8, 45.8-46.6)
	Qinghai-Tibet railway (Lhasa-Xining)	510	506	(99.3, 67.3-100)	479	470	(98.0, 64.8-99.9)
	Obskaya-Bovanenkovo railway	563	379	(67.2, 43.6-100)	563	373	(66.3, 31.2-100)
**RCP8.5 2061–2080**	Population	4,906,854	4,634,539	(94.5, 86.8-97.1)	4,567,438	1,287,285	(28.2, 23.6-43.7)
	Oil/Gas fields in the Russian Arctic	84,170	83,832	(99.6, 92.4-100)	83,870	57,350	(68.4, 60.2-91.4)
	TAPS (Prudhoe Bay-Valdez)	914	696	(76.2, 66.6-97.2)	913	496	(54.3, 31.7-97.9)
	Yamal-Nenets gas pipelines	1,341	1,341	(100, 100-100)	1,341	752	(56.1, 55.5-100)
	ESPO (Tayshet-Kozmino)	1,600	1,600	(100, 100-100)	1,600	741	(46.3, 45.8-49.1)
	Qinghai-Tibet railway (Lhasa-Xining)	510	510	(100, 96.5-100)	479	473	(98.8, 95.0-99.9)
	Obskaya-Bovanenkovo railway	563	563	(100, 100-100)	563	495	(87.8, 87.1-100)

TAPS = Trans-Alaska Pipeline System; ESPO = Eastern Siberia–Pacific Ocean pipeline
Oil/Gas fields = km^2; pipelines and railways = km

Supplementary Table 2. Numerical results of the infrastructure computations for periods 2041–60 and 2061–80 under three Representative Concentration Pathways (RCPs). Results are shown for the entire pan-Arctic permafrost area (Arctic) and its extracted subsets of Eurasia, North America, and central Asian mountains (Mountains). Proportion (%) of infrastructure in areas of near-surface permafrost thaw and high hazard (determined by a consensus of geohazard indices) appear in brackets.

		Thaw				High hazard			
	**Infrastructure**	**Arctic**	**Eurasia**	**North-America**	**Mountains**	**Arctic**	**Eurasia**	**North-America**	**Mountains**
**RCP2.6 2041–2060**	Settl./>5,000 inh.	1,155/39 (66.0/78.0)	755 (63.9)	162 (55.3)	238 (85.9)	556/22 (33.7/44.0)	427 (36.6)	70 (26.3)	59 (27.3)
	Buildings	90,766 (72.4)	83,943 (76.3)	3,904 (32.1)	2,919 (92.4)	32,221 (26.2)	29,883 (27.4)	1,965 (18.4)	373 (12.5)
	Roads	30,202 (68.6)	15,854 (61.8)	4,395 (65.0)	9,952 (85.9)	12,119 (28.4)	8,229 (32.1)	1,541 (23.1)	2,348 (22.6)
	Railways	5,728 (82.4)	4,806 (81.3)	340 (99.4)	581 (83.0)	2,956 (42.7)	2,335 (39.5)	227 (66.3)	394 (59.0)
	Airports	198 (51.8)	52 (49.1)	144 (52.7)	2 (66.7)	99 (27.7)	33 (32.4)	66 (26.0)	0 (0)
	Pipelines	5,921 (62.4)	5,234 (71.8)	686 (31.3)	1 (100)	2,703 (28.6)	2,422 (33.3)	282 (13.0)	0 (0)
	Industrial areas	81.5 (42.0)	79.5 (46.5)	1.5 (6.6)	0.5 (53.4)	44.9 (23.9)	43.6 (26.2)	1.3 (6.4)	<0.1 (0.1)
**RCP4.5 2041–2060**	Settl./>5,000 inh.	1,246/39 (71.2/78.0)	813 (68.8)	177 (60.4)	256 (92.4)	608/22 (36.9/44.0)	465 (39.8)	77 (28.9)	66 (30.6)
	Buildings	95,581 (76.3)	88,017 (80.0)	4,509 (37.1)	3,055 (96.7)	36,094 (29.4)	33,573 (30.7)	2,065 (19.3)	456 (15.3)
	Roads	33,366 (75.8)	17,754 (69.2)	4,902 (72.5)	10,710 (92.4)	13,584 (31.8)	9,176 (35.8)	1,765 (26.4)	2,643 (25.4)
	Railways	6,077 (87.4)	5,080 (85.9)	341 (99.5)	656 (93.7)	3,100 (44.8)	2,417 (40.9)	227 (66.5)	456 (68.3)
	Airports	220 (57.6)	60 (56.6)	157 (57.5)	3 (100)	111 (31.0)	38 (37.3)	73 (28.7)	0 (0)
	Pipelines	6,560 (69.2)	5,808 (79.6)	752 (34.3)	1 (100)	3,055 (32.4)	2,767 (38.1)	288 (13.3)	0 (0)
	Industrial areas	93.6 (48.2)	91.1 (53.3)	1.9 (8.6)	0.5 (53.4)	46.6 (24.8)	44.9 (27.1)	1.7 (7.9)	<0.1 (0.1)
**RCP8.5 2041–2060**	Settl./>5,000 inh.	1,351/44 (77.2/88.0)	897 (76.0)	191 (65.2)	263 (94.9)	665/23 (40.3/46.0)	518 (44.4)	81 (30.5)	66 (30.6)
	Buildings	106,748 (85.2)	96,991 (88.2)	6,657 (54.8)	3,100 (98.2)	39,840 (32.4)	36,339 (33.3)	3,037 (28.4)	464 (15.6)
	Roads	35,939 (81.7)	19,360 (75.4)	5,281 (78.1)	11,298 (97.5)	14,632 (34.3)	9,963 (38.9)	1,915 (28.7)	2,754 (26.5)
	Railways	6,732 (96.8)	5,695 (96.3)	342 (99.8)	694 (99.2)	3,423 (49.5)	2,708 (45.8)	227 (66.5)	488 (73.1)
	Airports	239 (62.6)	68 (64.2)	168 (61.5)	3 (100)	125 (34.9)	43 (42.2)	82 (32.3)	0 (0)
	Pipelines	7,699 (81.2)	6,913 (94.8)	785 (35.8)	1 (100)	3,759 (39.8)	3,258 (44.8)	501 (23.1)	0 (0)
	Industrial areas	126.4 (65.1)	123.3 (72.1)	2.6 (11.7)	0.5 (53.4)	57.6 (30.6)	55.3 (33.3)	2.2 (10.5)	<0.1 (0.1)
**RCP2.6 2061–2080**	Settl./>5,000 inh.	1,174/39 (67.0/78.0)	771 (65.3)	167 (57.0)	236 (85.2)	571/22 (34.6/44.0)	438 (37.5)	74 (27.8)	59 (27.3)
	Buildings	92,771 (74.0)	85,947 (78.1)	3,904 (32.1)	2,920 (92.5)	33,941 (27.6)	31,531 (28.9)	2,034 (19.0)	376 (12.6)
	Roads	30,855 (70.1)	16,370 (63.8)	4,557 (67.4)	9,927 (85.7)	12,515 (29.3)	8,578 (33.5)	1,598 (23.9)	2,339 (22.5)
	Railways	5,771 (83.0)	4,851 (82.1)	341 (99.5)	579 (82.8)	2,977 (43.0)	2,357 (39.9)	227 (66.5)	393 (58.9)
	Airports	206 (53.9)	55 (51.9)	149 (54.6)	2 (66.7)	104 (29.1)	35 (34.3)	69 (27.2)	0 (0)
	Pipelines	6,139 (64.7)	5,420 (74.3)	718 (32.8)	1 (100)	2,838 (30.1)	2,556 (35.1)	282 (13.0)	0 (0)
	Industrial areas	85.2 (43.9)	83.4 (48.8)	1.4 (6.5)	0.4 (41.3)	45.5 (24.2)	44.2 (26.6)	1.4 (6.4)	<0.1 (0.1)
**RCP4.5 2061–2080**	Settl./>5,000 inh.	1,342/44 (76.6/88.0)	891 (75.4)	188 (64.2)	263 (94.9)	663/23 (40.2/46.0)	517 (44.3)	80 (30.1)	66 (30.6)
	Buildings	106,332 (84.9)	96,931 (88.1)	6,302 (51.9)	3,099 (98.1)	39,816 (32.4)	36,314 (33.2)	3,039 (28.4)	463 (15.6)
	Roads	35,775 (81.3)	19,253 (75.0)	5,242 (77.5)	11,280 (97.3)	14,573 (34.1)	9,896 (38.6)	1,923 (28.8)	2,755 (26.5)
	Railways	6,687 (96.2)	5,652 (95.6)	341 (99.6)	694 (99.2)	3,391 (49.0)	2,676 (45.3)	227 (66.5)	488 (73.1)
	Airports	240 (62.8)	70 (66.0)	167 (61.2)	3 (100)	127 (35.5)	45 (44.1)	82 (32.3)	0 (0)
	Pipelines	7,690 (81.1)	6,904 (94.7)	785 (35.8)	1 (100)	3,845 (40.7)	3,260 (44.8)	586 (27.0)	0 (0)
	Industrial areas	124.5 (64.1)	121.5 (71.0)	2.5 (11.5)	0.5 (53.4)	56.2 (29.9)	53.9 (32.4)	2.4 (11.1)	<0.1 (0.1)
**RCP8.5 2061–2080**	Settl./>5,000 inh.	1,533/47 (87.5/94.0)	1,030 (87.2)	228 (77.8)	275 (99.3)	830/25 (50.3/50.0)	662 (56.7)	101 (38.0)	67 (31.0)
	Buildings	113,619 (90.7)	101,881 (92.6)	8,580 (70.6)	3,158 (100)	46,249 (37.6)	40,892 (37.4)	4,891 (45.7)	466 (15.7)
	Roads	39,266 (89.2)	21,892 (85.3)	5,800 (85.8)	11,574 (99.9)	17,359 (40.6)	12,158 (47.5)	2,427 (36.3)	2,774 (26.6)
	Railways	6,953 (100)	5,910 (100)	343 (100)	700 (100)	3,575 (51.7)	2,856 (48.4)	227 (66.5)	492 (73.7)
	Airports	286 (74.9)	83 (78.3)	200 (73.3)	3 (100)	173 (48.3)	60 (58.8)	113 (44.5)	0 (0)
	Pipelines	8,178 (86.2)	7,275 (99.8)	902 (41.2)	1 (100)	4,900 (51.9)	3,441 (47.3)	1,459 (67.3)	0 (0)
	Industrial areas	171.0 (88.1)	160.5 (93.9)	9.5 (42.8)	1.0 (100)	89.5 (47.6)	80.0 (48.2)	9.5 (44.9)	<0.1 (0.1)

Settl = settlements; inh. = inhabitants

Settlements, buildings, and airports = counts; roads, railways, and pipelines = km; Industrial areas = km^2

Supplementary Table 3. Numerical results of the proportion (%) and associated uncertainty range of infrastructure in areas of near-surface permafrost thaw and high hazard (determined by a consensus of geohazard indices) based on different Representative Concentration Pathway (RCP) scenarios by 2041–2060 and 2061–2080. The results are shown for the whole pan-Arctic permafrost area (Arctic) and its extracted subsets of Eurasia, North America and central Asian mountains (Mountains) (Settl = settlements and inh. = inhabitants).

Scenario	Infrastructure	Thaw: Arctic	Thaw: Eurasia	Thaw: North-America	Thaw: Mountains	High hazard: Arctic	High hazard: Eurasia	High hazard: North-America	High hazard: Mountains
RCP2.6 2041–2060	Settl./>5,000 inh.	66.0/78.0 (46.0-75.4/64.0-86.0)	63.9 (47.8-73.7)	55.3 (38.9-64.2)	85.9 (46.2-94.9)	33.7/44.0 (21.3-48.0/34.0-62.0)	36.6 (24.4-51.2)	26.3 (18.4-47.7)	27.3 (8.3-30.6)
	Buildings	72.4 (61.7-82.1)	76.3 (65.9-85.8)	32.1 (23.6-44.8)	92.4 (60.9-98.3)	26.2 (17.6-51.6)	27.4 (18.4-46.1)	18.4 (13.7-35.3)	12.5 (4.0-15.7)
	Roads	68.6 (42.9-80.8)	61.8 (43.1-74.4)	65.0 (43.2-76.7)	85.9 (42.3-97.1)	28.4 (16.2-40.5)	32.1 (20.5-45.4)	23.1 (13.7-42.2)	22.6 (7.4-27.3)
	Railways	82.4 (59.9-94.4)	81.3 (64.2-93.5)	99.4 (63.1-99.5)	83.0 (22.6-99.6)	42.7 (29.2-58.3)	39.5 (30.6-55.9)	66.3 (30.2-70.2)	59.0 (15.6-73.8)
	Airports	51.8 (35.6-61.5)	49.1 (34.0-60.4)	52.7 (36.3-61.5)	66.7 (33.3-100)	27.7 (17.0-44.7)	32.4 (18.6-53.9)	26.0 (16.5-41.3)	0 (0-0)
	Pipelines	62.4 (41.9-80.6)	71.8 (48.5-94.2)	31.3 (19.8-35.6)	100 (100-100)	28.6 (19.0-57.1)	33.3 (22.2-65.3)	13.0 (8.4-29.8)	0 (0-0)
	Industrial areas	42.0 (30.6-57.0)	46.5 (34.1-62.9)	6.6 (4.4-11.6)	53.4 (9.3-53.4)	23.9 (15.5-51.4)	26.2 (17.1-56.1)	6.4 (4.0-17.2)	0.1 (0-0.1)
RCP4.5 2041–2060	Settl./>5,000 inh.	71.2/78.0 (58.2-79.0/68.0-88.0)	68.8 (59.3-77.6)	60.4 (46.8-68.3)	92.4 (65.7-96.4)	36.9/44.0 (29.2-52.3/38.0-68.0)	39.8 (32.9-56.0)	28.9 (22.9-53.4)	30.6 (17.1-31.0)
	Buildings	76.3 (68.1-85.8)	80.0 (72.5-88.5)	37.1 (26.3-58.3)	96.7 (74.9-98.7)	29.4 (22.6-49.4)	30.7 (23.8-49.9)	19.3 (15.2-53.7)	15.3 (6.6-15.9)
	Roads	75.8 (56.8-83.3)	69.2 (55.5-77.3)	72.5 (57.2-80.4)	92.4 (59.3-98.5)	31.8 (23.0-43.6)	35.8 (28.2-48.7)	26.4 (19.3-49.4)	25.4 (12.8-27.6)
	Railways	87.4 (71.1-98.0)	85.9 (74.3-97.6)	99.5 (80.1-100)	93.7 (39.3-99.9)	44.8 (35.5-60.8)	40.9 (35.9-58.7)	66.5 (47.0-71.3)	68.3 (26.4-74.2)
	Airports	57.6 (45.5-66.2)	56.6 (44.3-67.9)	57.5 (45.8-65.2)	100 (66.7-100)	31.0 (23.7-51.7)	37.3 (28.4-62.7)	28.7 (22.0-47.6)	0 (0-0)
	Pipelines	69.2 (53.9-82.4)	79.6 (61.9-96.1)	34.3 (27.1-36.6)	100 (100-100)	32.4 (23.8-69.6)	38.1 (27.4-72.8)	13.3 (11.8-58.9)	0 (0-0)
	Industrial areas	48.2 (36.9-65.4)	53.3 (41.1-71.7)	8.6 (4.9-15.1)	53.4 (30.2-97.6)	24.8 (20.1-64.4)	27.1 (22.2-67.4)	7.9 (4.3-44.4)	0.1 (0.1-0.1)
RCP8.5 2041–2060	Settl./>5,000 inh.	77.2/88.0 (68.1-83.7/78.0-92.0)	76.0 (68.2-82.4)	65.2 (56.0-75.1)	94.9 (80.9-98.6)	40.3/46.0 (34.6-58.3/44.0-72.0)	44.4 (38.5-62.4)	30.5 (26.7-62.4)	30.6 (23.1-31.0)
	Buildings	85.2 (75.9-88.9)	88.2 (80.5-90.5)	54.8 (30.8-72.3)	98.2 (88.8-99.3)	32.4 (27.4-52.1)	33.3 (28.7-52.2)	28.4 (18.7-60.9)	15.6 (10.9-16.0)
	Roads	81.7 (71.3-86.7)	75.4 (69.1-81.7)	78.1 (67.2-83.7)	97.5 (78.6-99.6)	34.3 (29.6-48.9)	38.9 (35.4-53.8)	28.7 (23.2-63.0)	26.5 (19.6-27.8)
	Railways	96.8 (84.1-99.9)	96.3 (84.9-99.9)	99.8 (99.5-100)	99.2 (69.3-100)	49.5 (42.2-62.3)	45.8 (40.1-60.2)	66.5 (66.5-75.1)	73.1 (48.5-74.5)
	Airports	62.6 (54.2-71.5)	64.2 (55.7-74.5)	61.5 (53.5-70.0)	100 (66.7-100)	34.9 (28.2-63.7)	42.2 (36.3-73.5)	32.3 (25.2-60.2)	0 (0-0)
	Pipelines	81.2 (68.4-84.2)	94.8 (79.3-98.1)	35.8 (32.5-38.1)	100 (100-100)	39.8 (30.9-76.0)	44.8 (36.3-75.7)	23.1 (12.9-77.0)	0 (0-0)
	Industrial areas	65.1 (48.0-80.3)	72.1 (53.5-85.4)	11.7 (6.0-39.2)	53.4 (31.6-100)	30.6 (23.4-76.8)	33.3 (25.9-77.6)	10.5 (5.4-74.6)	0.1 (0.1-0.1)
RCP2.6 2061–2080	Settl./>5,000 inh.	67.0/78.0 (48.4-76.1/64.0-86.0)	65.3 (51.1-74.3)	57.0 (41.3-65.2)	85.2 (44.4-94.9)	34.6/44.0 (23.2-48.8/34.0-64.0)	37.5 (26.9-52.3)	27.8 (19.2-48.5)	27.3 (7.9-30.6)
	Buildings	74.0 (64.3-83.1)	78.1 (68.9-86.7)	32.1 (23.9-46.1)	92.5 (60.4-98.2)	27.6 (19.7-45.6)	28.9 (20.7-47.2)	19.0 (14.0-36.8)	12.6 (3.7-15.7)
	Roads	70.1 (45.6-81.3)	63.8 (46.6-75.0)	67.4 (48.7-77.6)	85.7 (41.6-97.4)	29.3 (17.7-41.6)	33.5 (22.5-46.7)	23.9 (15.6-44.2)	22.5 (7.1-27.3)
	Railways	83.0 (62.6-95.1)	82.1 (67.3-94.3)	99.5 (64.6-99.6)	82.8 (21.8-99.6)	43.0 (31.0-58.9)	39.9 (32.8-56.6)	66.5 (31.7-70.4)	58.9 (14.8-73.8)
	Airports	53.9 (38.5-62.8)	51.9 (35.8-61.3)	54.6 (39.6-63.0)	66.7 (33.3-100)	29.1 (19.0-45.3)	34.3 (20.6-55.9)	27.2 (18.5-41.3)	0 (0-0)
	Pipelines	64.7 (46.7-81.4)	74.3 (53.8-95.1)	32.8 (22.8-36.0)	100 (100-100)	30.1 (20.4-63.4)	35.1 (23.6-70.9)	13.0 (9.8-38.0)	0 (0-0)
	Industrial areas	43.9 (33.2-60.0)	48.8 (37.0-66.3)	6.5 (4.8-11.6)	41.3 (9.3-53.4)	24.2 (16.9-56.6)	26.6 (18.7-61.0)	6.4 (4.2-25.2)	0.1 (0-0.1)
RCP4.5 2061–2080	Settl./>5,000 inh.	76.6/88.0 (68.0-83.6/78.0-92.0)	75.4 (67.8-82.0)	64.2 (55.3-75.4)	94.9 (81.9-98.6)	40.2/46.0 (34.6-58.1/44.0-72.0)	44.3 (38.3-62.3)	30.1 (26.7-61.7)	30.6 (24.1-31.0)
	Buildings	84.9 (75.0-89.1)	88.1 (79.5-90.3)	51.9 (30.3-75.5)	98.1 (89.1-99.3)	32.4 (27.3-52.1)	33.2 (28.6-52.2)	28.4 (18.7-62.0)	15.6 (11.4-16.0)
	Roads	81.3 (70.7-86.5)	75.0 (68.3-81.3)	77.5 (66.2-83.5)	97.3 (78.7-99.6)	34.1 (29.4-48.4)	38.6 (35.0-53.5)	28.8 (23.0-60.9)	26.5 (19.8-27.8)
	Railways	96.2 (83.3-99.9)	95.6 (83.9-99.9)	99.6 (99.5-100)	99.2 (69.6-100)	49.0 (41.7-62.3)	45.3 (39.5-60.2)	66.5 (66.5-74.8)	73.1 (48.7-74.5)
	Airports	62.8 (53.7-72.0)	66.0 (54.7-74.5)	61.2 (53.1-70.7)	100 (66.7-100)	35.5 (27.9-62.6)	44.1 (35.3-73.5)	32.3 (25.2-58.7)	0 (0-0)
	Pipelines	81.1 (66.3-84.1)	94.7 (76.6-98.0)	35.8 (32.3-37.6)	100 (100-100)	40.7 (29.6-75.8)	44.8 (34.7-75.7)	27.0 (12.9-76.3)	0 (0-0)
	Industrial areas	64.1 (46.4-79.7)	71.0 (51.7-85.2)	11.5 (6.0-36.3)	53.4 (31.6-100)	29.9 (23.2-76.2)	32.4 (25.6-77.3)	11.1 (5.4-71.0)	0.1 (0.1-0.1)
RCP8.5 2061–2080	Settl./>5,000 inh.	87.5/94.0 (80.8-93.9/88.0-98.0)	87.2 (80.6-94.2)	77.8 (67.9-87.4)	99.3 (95.3-100)	50.3/50.0 (41.5-71.8/46.0-82.0)	56.7 (46.1-76.5)	38.0 (30.1-84.2)	31.0 (30.6-31.0)
	Buildings	90.7 (86.4-95.2)	92.6 (89.4-95.9)	70.6 (56.6-88.3)	100 (98.0-100)	37.6 (32.0-61.4)	37.4 (32.7-59.9)	45.7 (29.7-89.4)	15.7 (15.3-16.0)
	Roads	89.2 (84.1-93.0)	85.3 (79.4-89.4)	85.8 (79.9-94.8)	99.9 (96.8-100)	40.6 (35.1-61.8)	47.5 (40.8-67.7)	36.3 (27.0-92.2)	26.6 (26.0-27.8)
	Railways	100 (99.4-100)	100 (99.7-100)	100 (99.8-100)	100 (96.3-100)	51.7 (50.7-63.8)	48.4 (47.5-60.9)	66.5 (66.5-91.4)	73.7 (71.0-74.5)
	Airports	74.9 (66.0-86.4)	78.3 (71.7-87.7)	73.3 (63.4-85.7)	100 (100-100)	48.3 (33.8-85.8)	58.8 (45.1-84.3)	44.5 (29.5-87.0)	0 (0-0)
	Pipelines	86.2 (83.2-96.1)	99.8 (97.2-99.9)	41.2 (36.7-83.2)	100 (100-100)	51.9 (37.9-84.8)	47.3 (45.3-81.5)	67.3 (12.9-96.2)	0 (0-0)
	Industrial areas	88.1 (73.1-94.2)	93.9 (81.1-94.8)	42.8 (12.1-89.3)	100 (53.4-100)	47.6 (34.1-87.0)	48.2 (37.5-86.4)	44.9 (9.2-95.7)	0.1 (0.1-0.1)
