# Supplementary material for: Degrading permafrost puts Arctic infrastructure at risk by mid-century
Source: Nat Commun. 2018 Dec 11;9:5147. doi: 10.1038/s41467-018-07557-4 (PMC6289964; doi:10.1038/s41467-018-07557-4)
Supplement: Supplementary file 3 — Description of Additional Supplementary File [file 41467_2018_7557_MOESM3_ESM.pdf]

Description of Additional Supplementary File

Supplementary Data 1. Numerical results of the infrastructure computations using a consensus of geohazard indices (I_c) for periods 2041–2060 and 2061–2080 under three Representative Concentration Pathways (RCPs). The results are shown for the whole pan-Arctic permafrost area (Arctic) and its extracted subsets of Eurasia, North America and central Asian mountains (Mountains). Proportion (%) of infrastructure in areas of near-surface permafrost thaw and hazard class appear in brackets. The areal coverage of I_c is slightly smaller than that of modelled permafrost owing to the patchiness of sediment data in certain high-Arctic areas with thin overburden.
